# A Rare Case of Bicalutamide-Induced Severe Congestive Heart Failure in a Patient With Advanced Prostate Cancer

**DOI:** 10.7759/cureus.60298

**Published:** 2024-05-14

**Authors:** Ammar Y Abdulfattah, Salman Tajuddin, Nada Akkari, Omar I Elsayed, Suzette Graham-Hill

**Affiliations:** 1 Department of Internal Medicine, State University of New York Downstate Medical Center, Brooklyn, USA; 2 Internal Medicine, LewisGale Medical Center, Salem, USA; 3 Department of Cardiology, State University of New York Downstate Medical Center, Brooklyn, USA

**Keywords:** cardio-oncology, androgen deprivation therapy, nonsteroidal androgen receptor inhibitor, prostate cancer, cardiac toxicity, cardiomyopathy, bicalutamide, congestive heart failure

## Abstract

Bicalutamide, a nonsteroidal androgen receptor inhibitor, is an established therapeutic agent for advanced prostate cancer but is associated with severe cardiovascular side effects in rare cases. This case report discusses a rare occurrence of severe systolic congestive heart failure (CHF) in a 68-year-old male undergoing treatment for advanced prostate cancer with bicalutamide, without concurrent use of gonadotropin-releasing hormone antagonists. The patient presented with non-specific abdominal and bilateral foot pain. The initial assessment indicated anemia and severe dyspnea, revealing a significant decrease in left ventricular ejection fraction (LVEF) from 55% to 15% on transthoracic echocardiography (TTE), indicative of severe CHF. Bicalutamide was identified as the likely culprit given the temporal association and lack of other identifiable causes, leading to its discontinuation and initiation of guideline-directed medical therapy (GDMT).

A remarkable recovery of cardiac function was subsequently observed, with LVEF improving to 60%. The patient was managed with GDMT, and a gonadotropin-releasing hormone antagonist, degarelix, was later introduced for prostate cancer treatment, along with ongoing cardiac monitoring. The recovery of LVEF and the absence of other etiologies reinforce the likelihood of bicalutamide-induced cardiotoxicity. This report underscores the importance of vigilant cardiovascular monitoring in patients receiving bicalutamide, prompt identification of cardiac dysfunction and possible mechanisms of bicalutamide cardiotoxicity, and the potential for cardiac recovery upon drug discontinuation and initiation of GDMT.

## Introduction

Bicalutamide is an oral nonsteroidal androgen receptor inhibitor approved for the management of advanced prostate cancer. It can competitively inhibit the binding of androgens such as testosterone and dihydrotestosterone to androgen receptors [1]. While bicalutamide is a well-tolerated drug, it has been associated with certain adverse effects, the most common being endocrine-related (such as hot flashes, gynecomastia), neuropathic pain, gastrointestinal symptoms, and arthralgia; however, cardiovascular dysfunction related to bicalutamide treatment is under-reported [1]. There is a paucity of clinical data on the link between bicalutamide use and cardiovascular dysfunction, specifically congestive heart failure (CHF). Systemic cancer therapy is often associated with organ system dysfunctions and complications including the cardiovascular system, which may require acute care. Cancer therapy-related cardiac dysfunction (CTRCD) is commonly defined as a decrease in left ventricular ejection fraction (LVEF) of greater than 10%, to a value less than 53%, after the initiation of a cancer therapeutic agent. CTRCD is classified as either permanent and irreversible (type I) or reversible (type II) [2].

In this report, we present a rare case of bicalutamide-induced severe cardiac dysfunction leading to severe systolic CHF in a 68-year-old male with newly diagnosed advanced prostate cancer. Despite the generally accepted safety profile of bicalutamide, emerging evidence suggests a potential link between its use and cardiovascular complications. Given the significant impact of bicalutamide-induced cardiotoxicity, this case report highlights the importance of continued patient monitoring for adverse cardiovascular effects associated with bicalutamide therapy in prostate cancer patients. The report also emphasizes the need for careful cardiovascular risk assessment before initiating bicalutamide treatment and prompt discontinuation of the drug along with initiation of guideline-directed medical therapy (GDMT) when cardiac dysfunction occurs.

## Case presentation

The patient was a 68-year-old African-American male, with benign prostate hyperplasia, type 2 diabetes mellitus, chronic peripheral artery disease, and a recently diagnosed advanced prostate cancer undergoing treatment with androgen deprivation therapy (ADT) using bicalutamide. He presented to the emergency room with worsening bilateral foot pain and non-specific abdominal pain for two weeks. On physical examination, his vital signs were notable for tachycardia of 111 beats per minute, a tachypnea of 21 breaths per minute, blood pressure of 129/50 mmHg, temperature of 99 °F (37.2 °C), and oxygen saturation of 99% on room air; the remainder of the examination was unremarkable.

**Figure 1:**
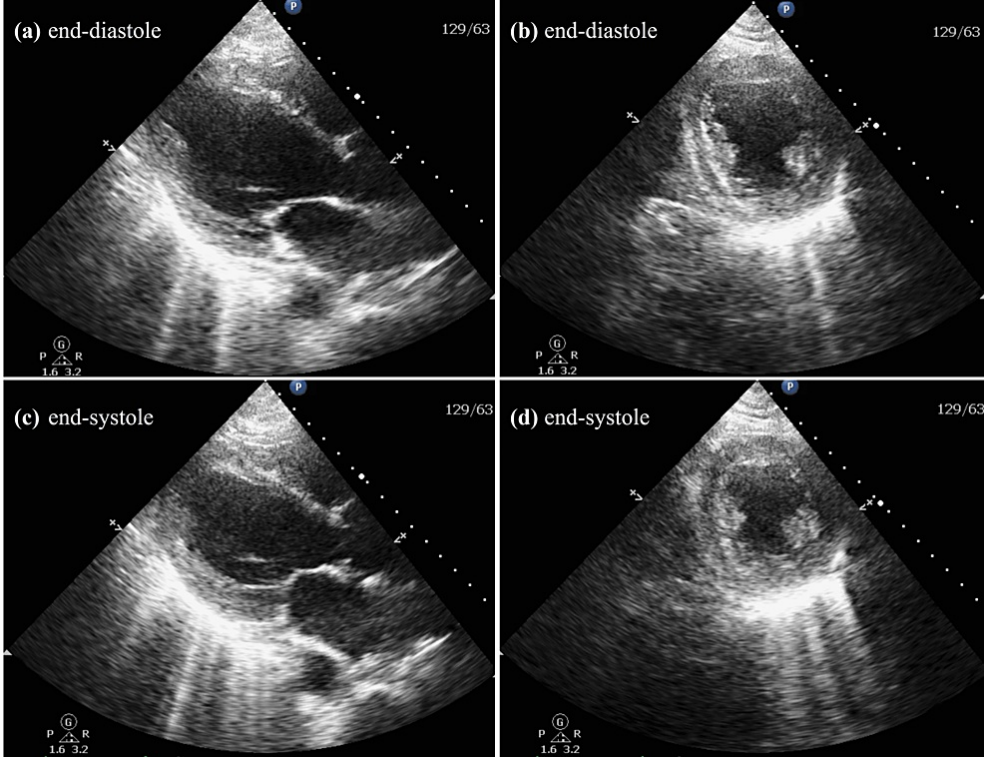
Transthoracic echocardiogram two months after the initiation of bicalutamide Transthoracic echocardiogram parasternal long axis and short axis views two months after the initiation of bicalutamide. (a) and (b): end-diastole long axis and short axis views, respectively; (c) and (d): end-systole long axis and short axis views, respectively. The left ventricular ejection fraction was 15% with severely decreased systolic function and mildly dilated left ventricle but normal left ventricular wall thickness

Initial laboratory findings were significant for a hemoglobin of 4.5 g/dL, a mean corpuscular volume of 100 femtoliters, and an alkaline phosphatase of 569 units per liter. A CT angiography (CTA) of the abdomen and pelvis was done to rule out any bleeding, which showed severe atherosclerosis of the abdominal aorta, left common iliac, and right superficial femoral arteries but no bleeding. The patient was urgently transfused with packed red blood cells. During the blood transfusion, the patient developed severe dyspnea with decreased oxygen saturation to 85%, tachypnea, and tachycardia. Transfusion-related acute lung injury (TRALI) and/or transfusion-associated circulatory overload (TACO) were suspected, and a chest X-ray was obtained, which revealed fluid overload. Laboratory tests showed pro-B-type natriuretic peptide (pro-BNP) significantly elevated to 2,307 pg/mL. The patient was given adequate diuresis with furosemide, leading to overall symptomatic improvement. However, the patient remained in sinus tachycardia to 115 beats per minute on electrocardiography. A transthoracic echocardiogram (TTE) was obtained and showed an LVEF of 15%, indicating severe CHF (Figure 1).

**Figure 2:**
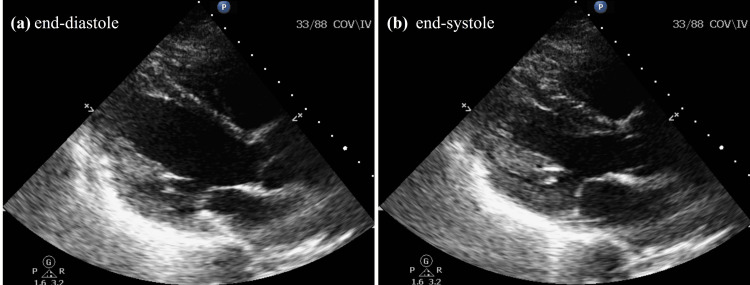
Transthoracic echocardiogram before the initiation of bicalutamide Transthoracic echocardiogram parasternal long axis view before the initiation of bicalutamide: (a) end-diastole and (b) end-systole. The left ventricular ejection fraction was 50-55% with normal left and right ventricular wall size and thickness

Two months before this presentation, the patient had been diagnosed with grade V prostate cancer with perineural invasion and pelvic osseous metastasis. He had been started on bicalutamide 50 mg orally once daily. No gonadotropin-releasing hormone (GnRH) antagonist had been started at that time. Before the initiation of bicalutamide therapy, the patient had undergone thorough evaluations including an initial laboratory test that showed a pro-BNP of 81 pg/mL, and a baseline TTE that found a LVEF of 55% with no wall motion abnormalities or ventricular dysfunction (Figure 2).

Management and follow-up

After consulting with the Oncology and Cardiology services, bicalutamide was discontinued, and GDMT including metoprolol succinate, sacubitril-valsartan, empagliflozin, and spironolactone was initiated. A tumor board meeting about starting the patient on any androgen deprivation therapy decided to continue to withhold treatment while the patient recovered from cardiomyopathy since GnRH medications such as degarelix or leuprolide had well-known cardiotoxic effects.

**Figure 3:**
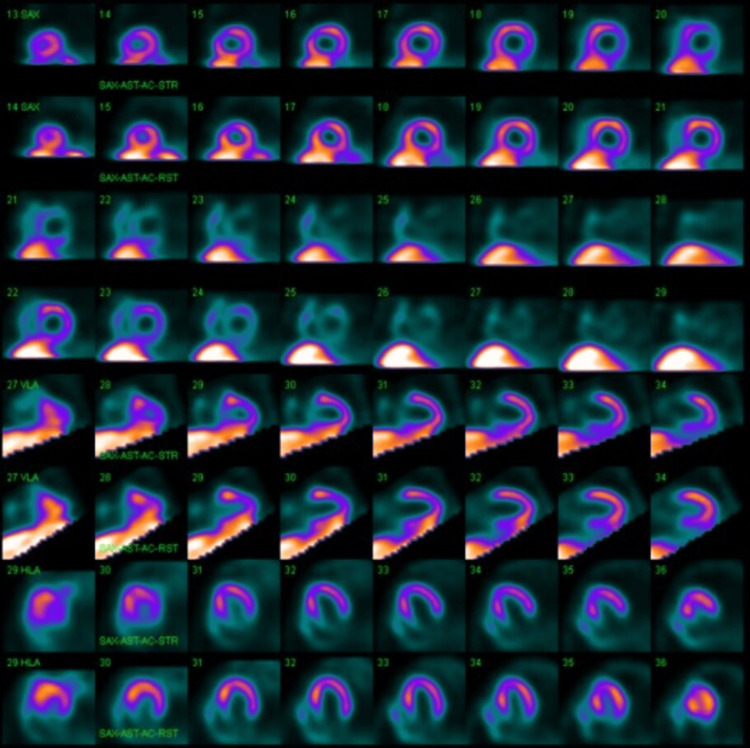
Results of a pharmacologic nuclear stress test The results showed no evidence of regional wall motion abnormalities or myocardial ischemia. The left ventricular ejection fraction was between 55% and 60%

The patient continued to improve on GDMT and was monitored closely in the Cardiology outpatient clinic. A month after discontinuing bicalutamide, a pharmacologic nuclear stress test was performed to rule out ischemic cardiomyopathy as well as help aid the decision regarding initiating prostate cancer therapy. The sestamibi myocardial perfusion scan revealed an LVEF between 55% and 65%, which is consistent with normal left ventricular function, and no evidence of regional wall motion abnormalities or myocardial ischemia (Figure 3). After a thorough risk-benefit discussion between the medical team and the patient, the decision was made to start ADT using the GnRH antagonist degarelix.

**Figure 4:**
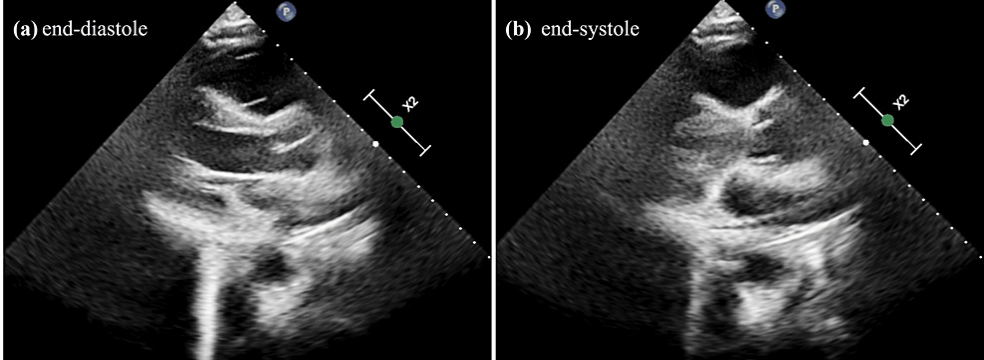
Transthoracic echocardiogram one month after the discontinuation of bicalutamide Transthoracic echocardiogram parasternal long axis view one month after the discontinuation of bicalutamide: (a) end-diastole and (b) end-systole. The left ventricular ejection fraction returned to the baseline value of 60%

During a subsequent follow-up in the Cardiology clinic a month later, the patient reported compliance with GDMT and denied any heart failure symptoms. A repeat TTE showed an LVEF of 60% with normal ventricular wall thickness, normal left ventricle size, and normal left ventricular systolic and diastolic functions (Figure 4). Despite the progression of the patient’s cancer, the LVEF remained within normal limits (LVEF=60%) four months after the discontinuation of bicalutamide. These findings collectively suggested that the severe CHF was likely induced by bicalutamide. The patient was closely monitored and followed up thereafter until he passed away due to complications of metastatic prostate cancer.

## Discussion

Prostate cancer is the leading cause of cancer diagnosis in men in the United States with an estimated incidence of 299,010 cases, and the second-leading cause of cancer death in men with an estimated 35,250 deaths in 2024 [3]. Prostate cancer is predominantly driven by androgen receptor signaling. In patients with advanced prostate cancer, ADT is used since androgen receptor signaling is one of the main molecular mechanisms behind prostate cancer progression [1]. ADT involves either a mechanical castration, also referred to as surgical ADT, which involves removal of the testes and spermatic cord resulting in a permanent testosterone depletion, or medical or chemical ADT, which involves chronic administration of GnRH antagonists such as degarelix or cetrorelix. ADT is usually the initial treatment in advanced prostate cancer patients [4]. When ADT, whether surgical or medical, is used in conjunction with a nonsteroidal androgen receptor inhibitor, it is referred to as combined androgen blockade (CAB). The effect of CAB is two-fold as it attempts to block androgen formation and androgen receptors [5,6].

Bicalutamide, a nonsteroidal androgen receptor inhibitor, is approved for use in combination with a luteinizing hormone-releasing hormone (LHRH) analog for the treatment of metastatic carcinoma of the prostate [7]. By competitively binding to the androgen receptors, bicalutamide functions as an antagonist blocking other androgens from binding to the receptors leading to androgen-dependent apoptosis of cells [8,9]. The usual starting dose of bicalutamide in prostate cancer, as in our patient, is 50 mg orally once daily. Regarding its adverse effects, bicalutamide is a well-tolerated drug and has a generally acceptable safety profile. Hot flashes, fatigue, back pain, arthralgia, constipation, diarrhea, and nausea are the most common adverse effects, reported at a rate of more than 10% [1]. More serious adverse effects have also been reported although much less frequently. The TERRAIN study - a randomized, double-blind, phase 2 study assessing the efficacy and safety of enzalutamide compared to bicalutamide in patients with metastatic prostate cancer - reported a 1% new-onset occurrence of CHF in the bicalutamide arm [1]. Dockery et al. reported a significantly increased proBNP level in prostate cancer patients who had received bicalutamide; compared to the baseline proBNP level, there was a significant linear increase in proBNP at three and six months of treatment [10].

Although a very rare adverse effect, CHF in prostate cancer patients on bicalutamide is a strong ground for discontinuation of the drug. However, a cause-and-effect relationship between bicalutamide and CHF could not be confirmed in the aforementioned study since the patients were already on ADT, which has been found to cause cardiovascular adverse effects including CHF [11]. Interestingly, the current patient was never started on any ADT, and hence his new-onset CHF symptoms were more likely due to bicalutamide since the symptoms only occurred following the initiation of bicalutamide. Moreover, the patient had no previous CHF symptoms or symptomatic cardiovascular pathology before the initiation of bicalutamide. The normal LVEF on the TTE performed before the initiation of bicalutamide further corroborates the potential causal relationship between bicalutamide and our patient’s new-onset CHF.

The mechanisms by which bicalutamide causes cardiac toxicity are poorly understood. Bicalutamide may cause cardiovascular adverse effects through hormonal imbalance, mainly low testosterone levels and subsequent metabolic effects [12]. One of the commonly investigated mechanisms is the androgen-androgen receptor (AR) effect on angiotensin II-induced vascular remodeling. Ordinarily, the AR system plays a protective role against vascular remodeling but it has been reported that angiotensin II stimulation markedly increased arterial medial thickness and perivascular and cardiac fibrosis in AR knock-out mice [13,14]. The deleterious effects of bicalutamide in antagonizing the AR system may contribute to vasodilatory failure. Vascular remodeling and vasodilatory failure are known to cause left ventricular dysfunction and consequently CHF [15].

Alteration in nitric oxide (NO) levels may be a potential mechanism for bicalutamide-induced cardiac toxicity. Ikeda et al. reported significantly reduced daily urinary NO metabolites excretion as a marker of NO bioavailability, aortic endothelial NO synthase expression and phosphorylation, and Akt phosphorylation regardless of angiotensin II stimulation in AR knock-out mice when compared with wild-type mice [14]. The decrease in NO, which can be achieved through the AR-antagonizing effect of bicalutamide, can lead to vascular remodeling [15]. On the other hand, an increase in endothelin-1 level via the endothelin A receptors was shown to decrease the NO level. Bicalutamide-mediated androgen deprivation causes increased endothelin A receptor expression and therefore endothelin-1 function [16,17]. This has been reinforced by the findings of Pflug et al., who reported that bicalutamide increases the secretion of endothelin-1 [18].

Another potential mechanism by which bicalutamide may cause cardiac dysfunction could be through the modulation of the transcription factor cyclin-A, which has been shown to induce myocardial regeneration and enhance cardiac function in ischemic heart failure [19]. Interestingly, bicalutamide can directly inhibit cyclin-A expression through the inhibition of fibroblast growth factor-8 (FGF-8) mRNA expression and FGF-8 protein secretion [20]. This direct inhibitory effect reveals the negative impact of bicalutamide on the myocardium and cardiac function. Therefore, through various cellular and molecular mechanisms, it is plausible that bicalutamide could lead to cardiac toxicity and subsequent cardiac dysfunction. This case report adds to the limited available data on the association between bicalutamide use in prostate cancer patients and the development of CHF. The fact that the cardiac function of our patient was reversible after the discontinuation of the drug suggests that CTRCD due to bicalutamide may have a type II pattern [2]. Further studies are required to gain deeper insights into bicalutamide-induced cardiac toxicity.

## Conclusions

We presented a rare case of bicalutamide-induced severe CHF (LVEF=15%) in an elderly male with newly diagnosed metastatic prostate cancer. Following the discontinuation of bicalutamide and initiation of GDMT, the patient’s cardiac function returned to his baseline (LVEF=55% to 60%) despite the progression of his prostate cancer. The reversible nature of the cardiac dysfunction suggests that bicalutamide-induced CTRCD may have a type II pattern. This care report underscores the necessity of a thorough evaluation of prostate cancer patients before the initiation of bicalutamide, and close follow-up while on treatment. Future studies are warranted to further characterize the pattern and impact of bicalutamide-induced cardiac toxicity and to further elucidate the underlying molecular mechanisms behind the deleterious effects of bicalutamide on the cardiovascular system.
